# Don’t forget to SMILE—a novel radiological index to characterize symptomatic minor instability of the lateral elbow

**DOI:** 10.1007/s00330-023-10259-1

**Published:** 2023-09-30

**Authors:** Jan-Peter Grunz

**Affiliations:** Department of Diagnostic and Interventional Radiology, University Hospital Würzburg, Oberdürrbacher Straße 6, 97080 Würzburg, Germany

Lateral elbow pain constitutes a common cause of presentation to emergency departments and orthopedic surgery units worldwide [1]. While the majority of patients are diagnosed with lateral epicondylitis (“tennis elbow”), tendinopathy is not the only condition responsible for the characteristic symptoms [2]. To account for this observation, lateral elbow pain was redefined as a multifactorial process in recent years, comprising extra-articular, intra-articular, and systemic aspects [3]. One particular factor gaining increasing recognition is the development of micro-instability caused by patholaxity of the ligamentous stabilizers of the lateral elbow [4]. In contrast to previous considerations, epicondylitis appears to be merely the result of secondary stabilizer overuse to compensate for the true underlying cause, i.e., functional incompetence of the lateral collateral ligament complex and in particular the annular ligament. This condition has been investigated arthroscopically before, leading to the authors establishing the term “symptomatic minor instability of the lateral elbow” (SMILE) [5].

Similar to other elbow pathologies, diagnostic imaging precedes surgical evaluation in the vast majority of patients with SMILE. As a result, standardization of radiological assessment is essential to analyze the severity of instability in reliable fashion. In their article “Semiquantitative index of symptomatic minor instability of the lateral elbow at CT arthrography (SMILE index): clinical applicability and reproducibility study,” Zagarella et al propose a novel radiological index that considers different aspects of SMILE, i.e., chondromalacia, annular ligament laxity, synovial thickening, humeroradial joint asymmetry, and capsular tears. A maximum of 8 points can be attributed in total, allowing for a fine-granular classification of minor instability [6]. Interestingly, the SMILE index was conceptualized and investigated based on the detailed analysis of 90 CT arthrograms in 80 individuals with ultrasound-guided articular injection of contrast agent. While MRI is certainly the more common imaging modality to diagnose potential causes of lateral elbow pain [7], CT-based evaluation offers a plethora of advantages, such as shorter examination times, precise evaluation of concomitant bone pathologies, less contraindications, and even superior positioning options if modern cone-beam CT scanners are used [8].

Based on substantial inter-reader agreement between an expert radiologist with 15 years of clinical experience and a radiology resident (Cohen’s *κ* = 0.75, concordance of 67%) as well as excellent intra-reader reliability in repeated assessment by the expert observer (*κ* = 0.94, concordance of 87%), the authors postulate that the SMILE index can support treatment decision-making by providing orthopedic surgeons with reproducible radiological analyses. With an application time of less than 4 min, the index appears compatible with the tight reading schedule of most radiology departments, suggesting good applicability for daily clinical routine.

Further investigations are certainly warranted to determine whether the proposed index also translates to MRI or MR arthrography and to what extent the imaging findings correlate with elbow arthroscopy. However, for radiologists currently struggling with the systematic evaluation of lateral elbow pain, it may be best to welcome CT arthrograms with a SMILE in the future.

## Declarations

### Guarantor

The scientific guarantor of this publication is Jan-Peter Grunz (Grunz_J@ukw.de).

### Statistics and biometry

No complex statistical methods were necessary for this paper.

### Methodology

- • Commentary
